# Crescentic transformation induced by granulocyte-colony stimulating factor administration—a case report and review of the literature

**DOI:** 10.1093/ckj/sfag258

**Published:** 2026-08-11

**Authors:** Hariharan Seshadri, Yassmin Falahat, Waseem Abdelrahim, Yimin Geng, Amanda Tchakarov, Ala Abudayyeh

**Affiliations:** Section of Nephrology, Division of Internal Medicine, The University of Texas MD Anderson Cancer Center, Houston, USA; Section of Nephrology, Division of Internal Medicine, The University of Texas MD Anderson Cancer Center, Houston, USA; Section of Nephrology, Division of Internal Medicine, The University of Texas MD Anderson Cancer Center, Houston, USA; Research Medical Library, The University of Texas MD Anderson Cancer, Houston, USA; Department of Pathology and Laboratory Medicine, The University of Texas Health Science Center McGovern Medical School, Houston, USA; Section of Nephrology, Division of Internal Medicine, The University of Texas MD Anderson Cancer Center, Houston, USA

**Keywords:** crescentic glomerulonephritis, filgrastim, granulocyte-colony stimulating factor, onconephrology

## Abstract

The use of recombinant human granulocyte-colony stimulating factor (G-CSF) is an uncommon aetiology for acute kidney injury in cancer patients. Several patterns of renal injury due to G-CSF have been discussed in the literature. Here, we present a case of a 48-year-old man with monoclonal gammopathy of renal significance who received filgrastim for planned autologous stem cell transplantation and developed acute kidney injury presenting as crescentic glomerulonephritis. The patient was treated with steroids, plasmapheresis, and cyclophosphamide. He attained complete recovery and proceeded with stem cell transplantation. We conducted an extensive review of the literature on the subject, which revealed that 10 of the 18 biopsy-proven cases of G-CSF-induced renal injury presented as crescentic glomerulonephritis. When G-CSF is administered to patients with an underlying renal pathology (usually autoimmune or monoclonal in aetiology), the migration and degranulation of activated neutrophils in the glomerular microenvironment in large numbers results in glomerular basement membrane rupture and crescent formation. Timely renal biopsy and aggressive initiation of treatment aid in attaining renal recovery and a favourable prognosis in cancer patients.

## INTRODUCTION

Recombinant human granulocyte-colony stimulating factor (G-CSF) is commonly used to mobilize stem cells for transplant and in the management of neutropenia. Although it is generally regarded safe, rare immune-mediated adverse effects have been reported, affecting the cardiopulmonary, renal, and musculoskeletal systems. Renal involvement can range from mild proteinuria and haematuria to persistent glomerular injury, often in patients with a pre-existing renal disease. Although data on the incidence of G-CSF-related nephrotoxicity are limited, the Severe Chronic Neutropenia International Registry reported 25 cases of glomerulonephritis and/or persistent haematuria among 853 patients (2.9%) on chronic filgrastim therapy [1]. Several patterns of G-CSF-induced renal injury have been previously documented in the literature. Here, we report a case of crescentic glomerulonephritis in a patient with monoclonal gammopathy of renal significance (MGRS) following G-CSF administration. We also present a comprehensive review of the literature, through which we have identified the common patterns of renal injury and their clinical management and prognostic outcomes.

## CASE PRESENTATION

A 48-year-old Caucasian man was referred to our institution for the management of biopsy-proven MGRS with monoclonal immunoglobulin G (IgG) kappa and C3 deposits presenting with membranoproliferative glomerulonephritis (MPGN). He was previously treated with bortezomib, cyclophosphamide, and dexamethasone, and autologous stem cell transplant had been planned. His medical history was significant for hypertension and hyperlipidaemia. Physical examination revealed bilateral pedal oedema, while other systems were unremarkable. Baseline laboratory tests showed a serum creatinine of 1.6 mg/dl, white blood cell (WBC) count of 8100/mm^3^ (neutrophils: 2820/mm^3^), and urinalysis with 3+ proteinuria and no red blood cells. Ultrasonography of the kidneys and urinary tract was unremarkable. There was no history of infections in the month prior to this visit, and routine viral markers were negative.

After a thorough evaluation, a single dose of G-CSF, filgrastim, was administered subcutaneously at a dose of 1080 μg (∼10 µg/kg) to facilitate stem cell mobilization. The next day, the patient noticed the passage of dark-coloured urine. Repeat investigations revealed an increase in serum creatinine from 1.6 mg/dl to 6.7 mg/dl and elevated WBC counts (69 500/mm^3^, neutrophils: 25 520/mm^3^). Urinalysis showed protein >500 mg/dl, 141 red cells/field, 54 white cells/field, and hyaline casts, and 24-hour urine protein was 8 g/day. Given the acute kidney injury (AKI), stem cell apheresis was postponed and renal biopsy was performed.

**Figure 1:**
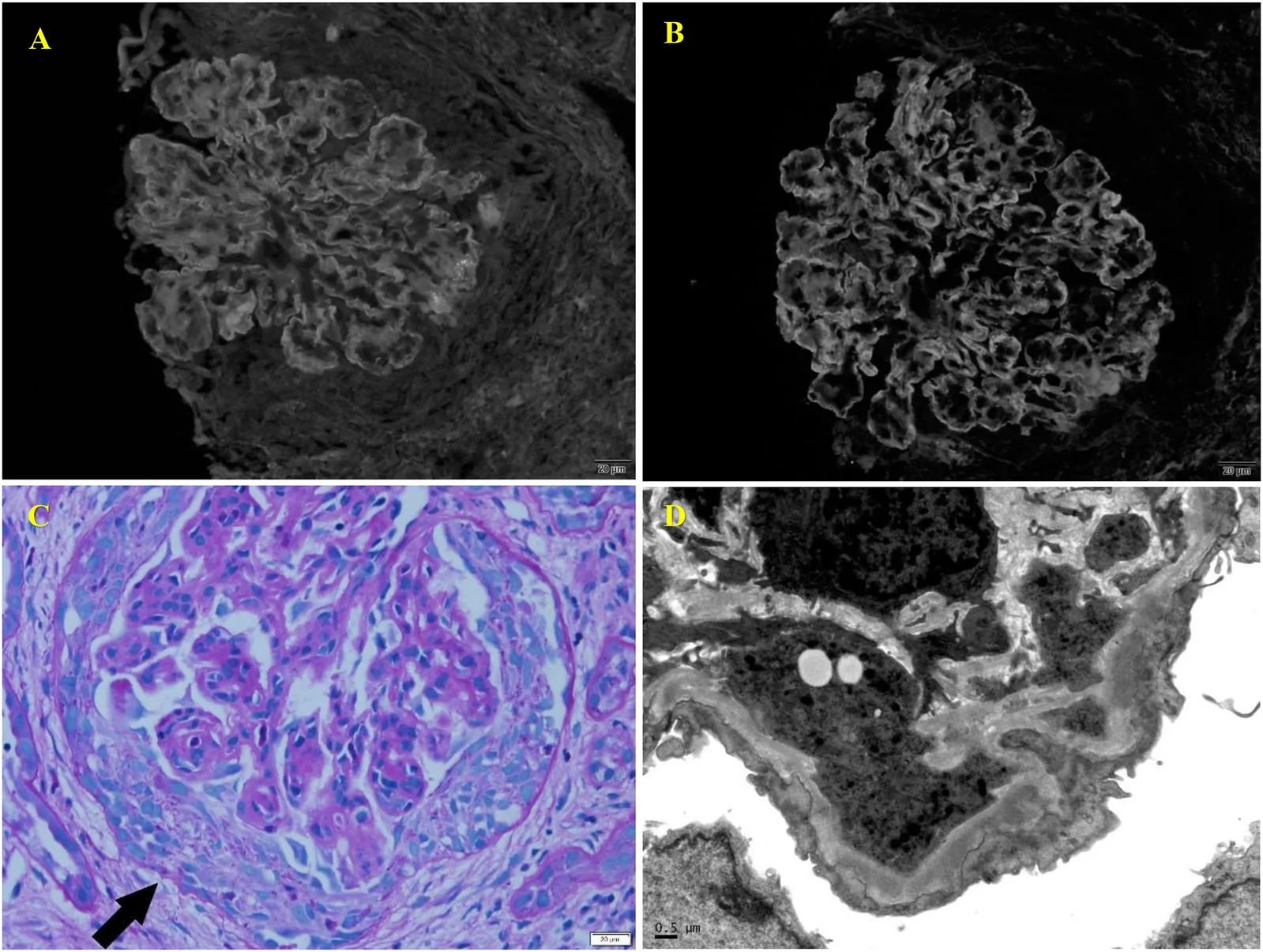
Renal biopsy specimen microscopy. Immunofluorescence images show global kappa light chain (A) and IgG (B) smudgy to slightly granular deposits (1+) in the capillaries. Light microscopy (C) with PAS Periodic acid-Schiff staining shows glomerulus with membranoproliferative pattern and cellular crescent (indicated with arrow). Electron microscopy shows sub-epithelial electron-dense deposits (D).

Histopathological examination revealed crescentic MPGN with monoclonal IgG kappa deposits with cellular crescents present in 62% (13/21) of the viable glomeruli. Mild arteriolar sclerosis, global sclerosis of 9% (2/23) of glomeruli, and diffuse moderate interstitial fibrosis (with ∼40% severe interstitial fibrosis) with tubular atrophy were also noted. Electron microscopy showed sub-epithelial electron-dense deposits (Fig. 1).

A comprehensive serological evaluation for secondary causes of crescentic glomerulonephritis was performed. Anti-neutrophil cytoplasmic antibodies (ANCAs) and anti-glomerular basement membrane antibodies were negative, with no clinical or laboratory evidence suggestive of systemic autoimmune disease or active infection. Complement evaluation and hematologic workup were consistent with the patient’s known MGRS-associated immune-complex-mediated MPGN.

Possible explanations for the acute renal injury were considered. The patient had stable chronic MGRS-associated MPGN without active nephritic manifestations prior to filgrastim exposure, making spontaneous crescentic transformation less likely. Treatment-related immune activation was considered less probable given the patient’s prior tolerance to therapy, while infection-related glomerular injury was considered unlikely in the absence of clinical or laboratory evidence of infection. The abrupt onset of gross haematuria and severe AKI immediately following filgrastim administration, together with marked leucocytosis and previously reported biopsy-proven cases of G-CSF-associated crescentic GN, supports filgrastim as the most likely precipitating factor for crescentic transformation in a predisposed glomerular microenvironment. Application of the Naranjo Adverse Drug Reaction Probability Scale yielded a score of 4, consistent with a possible drug-related adverse event (Supplementary Table S1).

**Figure 2:**
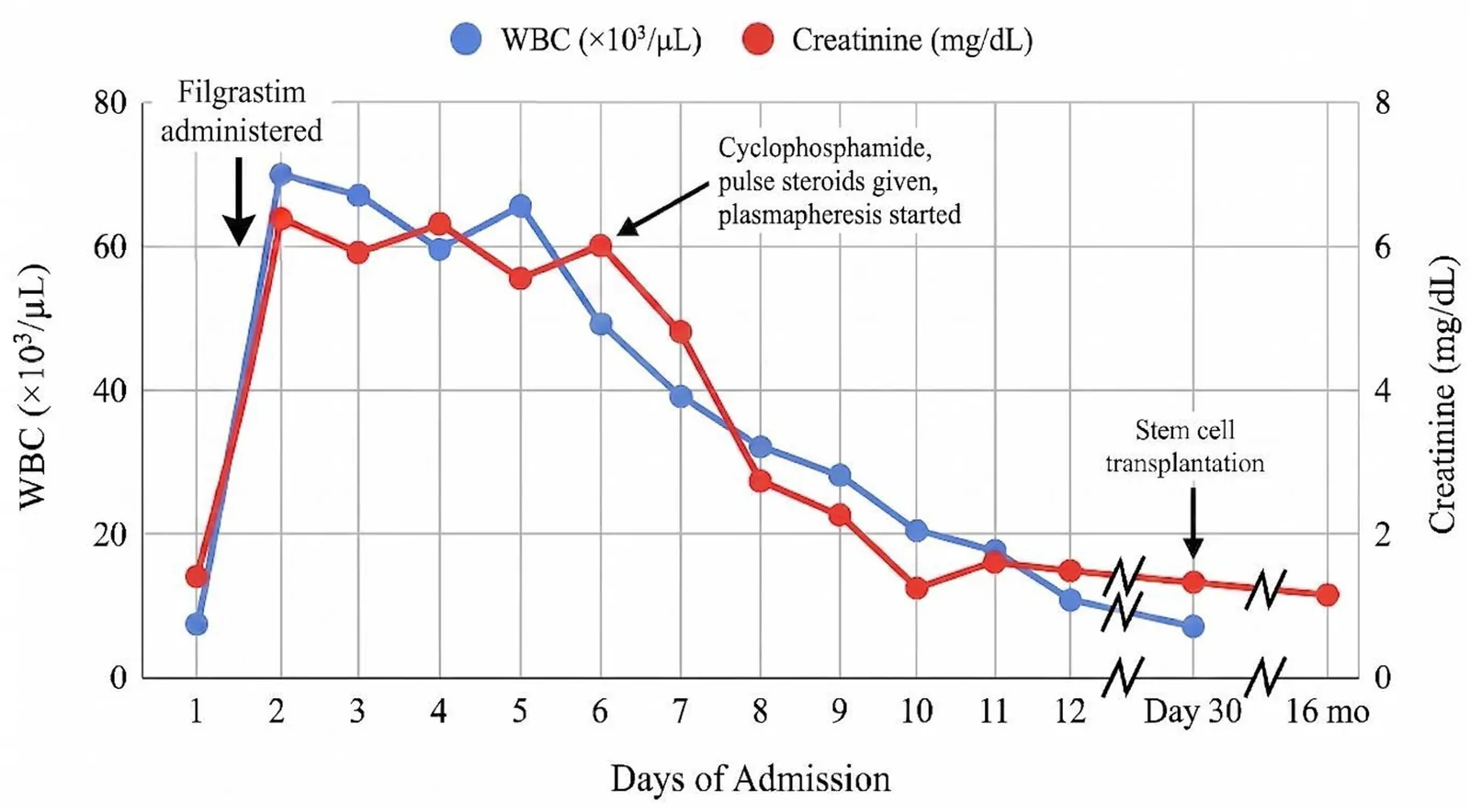
Creatinine and WBC trends during hospitalization. G-CSF (filgrastim) was administered on day 1. An acute increase in creatinine and WBC levels was noted by day 2. Cyclophosphamide, pulse steroids, and plasmapheresis were initiated on day 6. Recovery of renal function noted in further days. Stem cell transplantation was done after a month and renal recovery continued.

The patient received five sessions of plasmapheresis, a single dose of intravenous cyclophosphamide at a renal-adjusted dose of 0.5 g/m^2^, and two sessions of haemodialysis. Pulse steroid therapy (methylprednisolone 500 mg every 6 hours) was given for 3 days, after which the patient was transitioned to prednisone 60 mg and then discharged on a taper. The recovery process was uneventful, with good improvement in renal function and urine output (Fig. 2).

**Figure 3:**
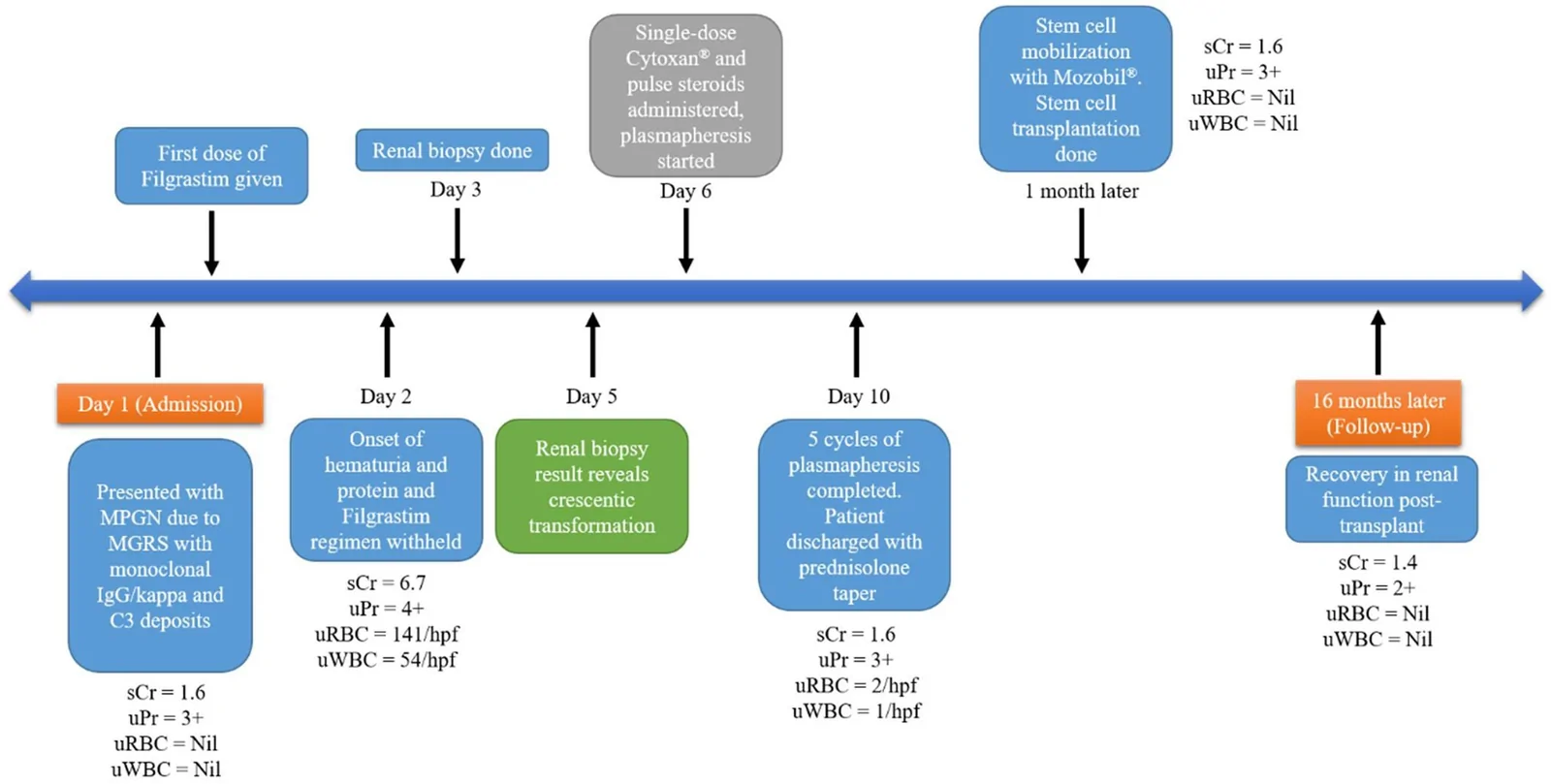
Clinical timeline of the patient. sCr, serum creatinine; uPr, urine protein; uRBC, urine red blood cells; uWBC, urine white blood cells; hpf, high-power field; cytoxan, cyclophosphamide; mozobil, plerixafor.

After 1 month, the patient was scheduled for stem cell mobilization after treatment with two doses of plerixafor 1080 mg (∼10 mg/kg). The apheresis yielded 9.95 million CD34+ cells/kg, after which the patient proceeded to stem cell transplant. His renal function continued to improve, and 16 months after transplant, his serum creatinine was down to 1.4 mg/dl and 24-hour urine protein from 8 g/day to 2 g/day (Fig. 3).

## LITERATURE REVIEW

As the incidence of G-CSF-induced kidney injury is rare, we reviewed the literature to further understand the spectrum of injury, treatments, and renal outcomes. We used a search strategy (1946–1 October 2025) we developed with a health information specialist (Y.G.) to search MEDLINE/Ovid, Embase/Ovid for cases of patients treated with G-CSF or granulocyte stimulating factor who developed kidney injury and/or glomerulonephritis. The search details and the terms used are included in the Supplementary materials (Supplementary Table S2). Three co-authors (A.A., H.S., and Y.F.) screened all 29 articles, excluding articles that did not contain renal outcomes and treatments. For each patient reported, we extracted relevant data, including demographic data, disease diagnosis data, treatment characteristics, laboratory data, type of kidney injury, immunosuppressive treatment characteristics, and renal outcomes. Renal response was defined as no response (no improvement in creatinine after treatment), partial response (any decrease in creatinine after treatment), and complete response (creatinine returning to baseline after treatment). Complete, partial, and stable proteinuria responses (evaluated at the time of renal recovery) were defined as a spot urine protein-to-creatinine ratio ≤200 mg/g (or 0.2 g/24 h), a spot urine protein-to-creatinine ratio <2 g/g (or 2 g/24 h), and no change in proteinuria, respectively.

From our review, the median patient age was 36 (25.5–51.5) years with a male-to-female ratio of 2.5:1. Filgrastim accounted for the highest proportion of reported renal injury cases (55.6%), followed by lenograstim (16.7%), pegfilgrastim (11.1%), and tbo-filgrastim (5.6%). This distribution may reflect the more widespread clinical use of filgrastim rather than true differences in nephrotoxic risk between formulations (owing to their differences in pharmacokinetic profiles). Renal injury has been noticed after the administration of a median of 4 (3–5.5) doses of G-CSF, with a median interval of 3.5 (2.8–6.5) days from the last dose to the onset of clinical symptoms. Among these cases, only 18 patients had undergone a renal biopsy to ascertain the pattern of injury (Table 1). Of these patients, 10 had crescentic glomerulonephritis, 4 had MPGN, 3 had IgA nephropathy, and 1 had acute tubular necrosis. The management of G-CSF-induced glomerulonephritis included immediate cessation of any further doses of G-CSF, timely renal biopsy to identify the nature of renal injury, and prompt initiation of appropriate immunosuppressive regimen. Among the 10 patients with crescentic glomerulonephritis, steroids and cyclophosphamide therapy was initiated in 4 patients, of which 2 were started on plasmapheresis (4–5 cycles) as well. In two patients, steroids alone were used as the mainstay treatment. Methylprednisolone 500–1000 mg was given as the pulse steroid therapy in most cases for 2–3 days, then transitioned to prednisone 60–80 mg, followed by a taper. Azathioprine (50 mg/day) and rituximab (1 g, two doses) were some other immunosuppressive agents used along with steroids in some cases. Haemodialysis was carried out in 3 of the 10 patients. It is interesting to note that in two patients, a good renal response was noted without any active intervention except the discontinuation of the G-CSF regimen. However, both these patients were altruistic donors and did not have any active malignancy. Overall, eight patients had complete or partial renal responses at the end of the treatment course.

**Table 1:**
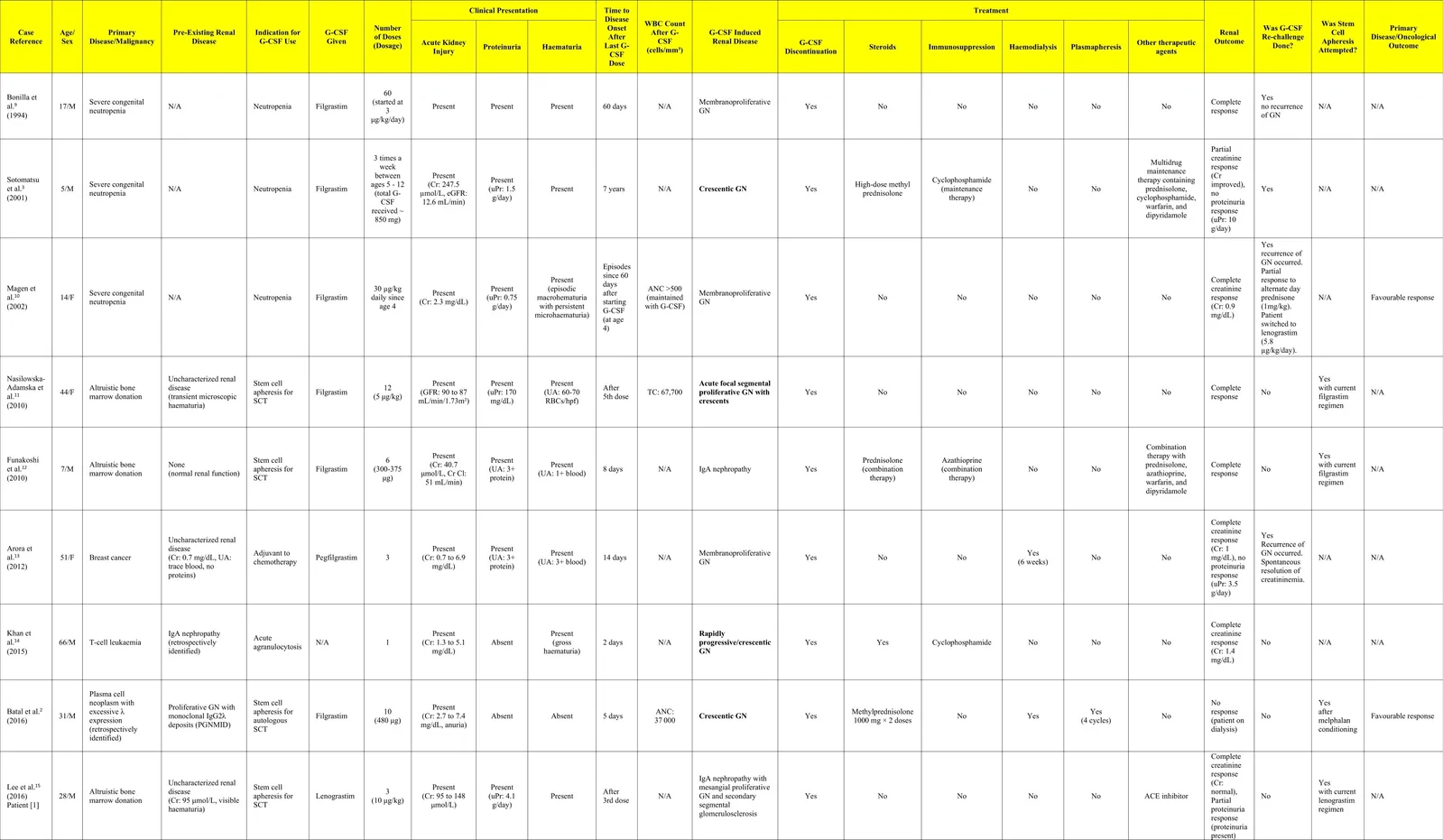

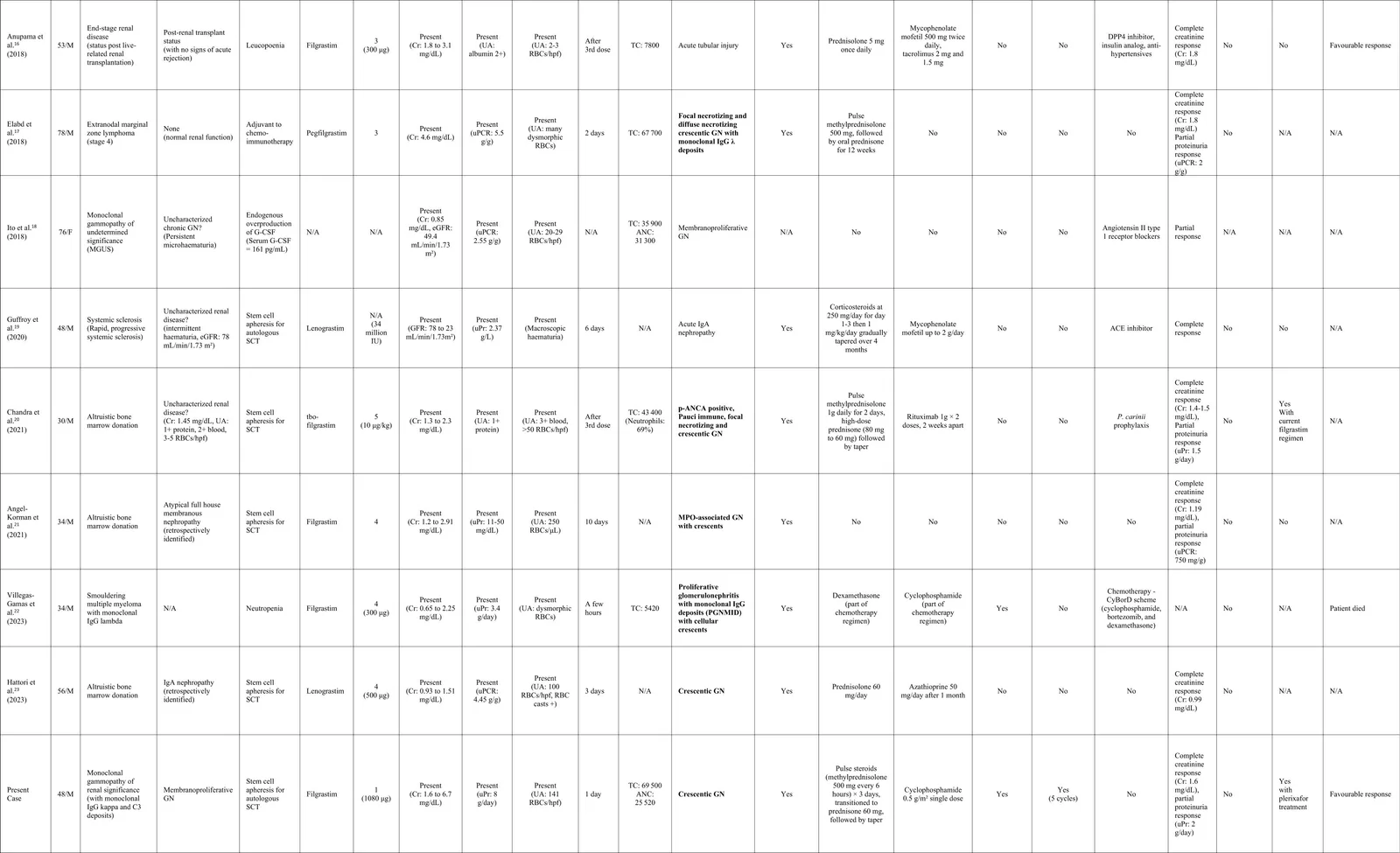
Literature Review of Biopsy-Proven Cases of G-CSF Induced Acute Kidney Injury.

Cases of crescentic glomerulonephritis are shown in bold.

G-CSF, granulocyte-colony stimulating factor; GN, glomerulonephritis; N/A, not available/applicable; SCT, stem cell transplant; WBC, white blood cells; RBC, red blood cells; hpf, high-power field; Cr, serum creatinine; UA, urine analysis; eGFR, estimated glomerular filtration rate; Cr Cl, creatinine clearance; uPr, urine protein; uPCR, urine protein-creatinine ratio; TC, total WBC count; ANC, absolute neutrophil count; ACE, angiotensin-converting enzyme; M, male; F, female

Stem cell apheresis was successfully attempted after completion of therapy in four of the six initially planned patients with crescentic glomerulonephritis. Among the two cases with reported outcomes, stem cell transplantation led to favourable oncological prognosis in both (Batal *et al*. [2] and the present case). G-CSF was re-challenged and continued in a patient with congenital neutropenia to prevent future infections under a multidrug maintenance therapy consisting of prednisolone and cyclophosphamide (Sotomatsu *et al*. [3]).

## DISCUSSION

Acute renal failure is a common complication in cancer patients. Deterioration of kidney function in these patients can be due to the malignancy per se (infiltration, obstruction, and paraneoplastic syndrome), the adverse effects of treatment (drug nephrotoxicity and tumour lysis), or a multitude of secondary factors (electrolyte imbalances, dehydration, sepsis, and comorbid illnesses) [4]. G-CSF-mediated renal injury constitutes a rare faction of aetiologies for kidney disease in cancer patients. In this report, we sought to highlight G-CSF use as a potential differential diagnosis for crescentic AKI and underscore the treatment and prognosis of such clinical scenarios.

G-CSF is a cytokine that is a component of acute phase inflammatory reactions. Under normal circumstances, the circulating level of G-CSF is under 40 pg/ml. However, this level can exceed 2000 pg/ml in times of physiological stress [5]. G-CSF augments neutrophil production, accelerates the maturation and release of neutrophils from the bone marrow, and amplifies neutrophilic chemotaxis and phagocytosis. G-CSF receptors are present on the cells of myeloid lineage, platelets, vascular endothelial cells, and lymphoid cells. G-CSF induces the expression of adhesion molecules (L-selectin, E-selectin, and ICAM, intercellular adhesion molecule-1) and their ligands on the target cells, thus promoting leukocyte–endothelium interactions [6]. Recombinant human G-CSF has been successfully expressed in *Escherichia coli* and produced in large quantities for the treatment of neutropenia and for aiding in stem cell apheresis.

Renal injury due to G-CSF can present in several forms, ranging from glomerular to tubulointerstitial involvement. The proposed pathogenesis of G-CSF-induced crescentic glomerulonephritis is a two-step process. When an individual with an underlying kidney disease (first hit) is administered G-CSF (second hit), the resultant neutrophilia and endothelial activation cause overt renal damage. This is because the localized deposition of immunoglobulins and complements in the diseased glomeruli attracts the activated neutrophils into the glomerular microenvironment. Heightened neutrophilic infiltration and degranulation results in the rupture of the glomerular basement membrane, leading to the formation of cellular crescents. The support for this two-hit hypothesis for the crescentic transformation is displayed in our literature review results, where in 8 of the 10 patients with crescentic glomerulonephritis, an underlying kidney disease was noted: three were associated with myeloma or a monoclonal process, one had IgA nephropathy, and four were from donor patients (one had IgA nephropathy, another had membranous nephropathy, and two patients had an uncharacterized renal disease). This is in line with the findings of Dale *et al*. from the Severe Chronic Neutropenia International Registry: of the 25 cases of glomerulonephritis reported due to G-CSF use, 12 patients had an underlying renal disease, and another 4 patients had baseline renal abnormalities [1].

Our literature review also indicates that crescentic transformation is likely driven by an underlying antibody-mediated process. Out of the 10 crescentic transformations, 8 had kidney-biopsy-proven light chain disease or associated immune deposition such as IgA or membranous nephropathy. However, the remaining two patients apparently had no underlying antibody-mediated process, namely, extranodal marginal zone lymphoma and congenital neutropenia.

It thus becomes evident that timely renal biopsy and aggressive treatment of G-CSF-induced renal injury helps to reverse the damage and prevent progression into chronic kidney disease. This not only helps improve renal function but also keeps the window open for exploring multiple therapeutic options in cancer patients, in whom overall survival depends on the fine balance between progression of renal dysfunction and oncological recovery [7, 8].

## CONCLUSION

This case highlights G-CSF as a potential trigger for abrupt crescentic transformation in previously stable MGRS-associated MPGN and demonstrates successful management with prompt diagnosis and aggressive treatment, without compromising on subsequent autologous stem cell transplantation. Our literature review identified a similar pattern, particularly among patients with autoimmune disorders or monoclonal/light-chain-mediated diseases. G-CSF-associated renal injury should therefore be considered in cancer patients who develop renal dysfunction. Baseline renal assessment, close monitoring after G-CSF administration, and early renal biopsy when indicated are essential, as timely intervention may halt disease progression, reverse renal injury, and improve outcomes.

## Supplementary Material

sfag258_Supplemental_File

## Data Availability

All data generated or analyzed during this study are included in this published article.

## References

[1] Dale DC, Cottle TE, Fier CJ et al. Severe chronic neutropenia: treatment and follow-up of patients in the Severe Chronic Neutropenia International Registry. Am J Hematol. 2003;72:82–93. 10.1002/ajh.10255 12555210

[2] Batal I, Markowitz GS, Wong W et al. Filgrastim-induced crescentic transformation of recurrent IgG2λ GN. J Am Soc Nephrol. 2016;27:1911. 10.1681/ASN.2016010061 27147425 PMC4926993

[3] Sotomatsu M, Kanazawa T, Ogawa C et al. Complication of rapidly progressive glomerulonephritis in severe congenital neutropenia treated with long-term granulocyte colony-stimulating factor (filgrastim). Br J Haematol. 2000;110:234–5. 10.1046/j.1365-2141.2000.02072-1.x 10931006

[4] Darmon M, Ciroldi M, Thiery G et al. Clinical review: specific aspects of acute renal failure in cancer patients. Crit Care. 2006;10:211. 10.1186/cc4907 16677413 PMC1550893

[5] Nishida M, Hamaoka K. How does G-CSF act on the kidney during acute tubular injury?. Nephron Exp Nephrol. 2006;104:e123–8. 10.1159/000094962 16902315

[6] Biologic and Molecular Effects of Granulocyte Colony-Stimulating Factor in Healthy Individuals: Recent Findings and Current Challenges. https://ashpublications.org/blood/article/111/4/1767/133205/Biologic-and-molecular-effects-of-granulocyte (7 February 2026, date last accessed). 10.1182/blood-2007-07-097543 18057230

[7] Karam S, Rosner MH, Sprangers B et al. Cancer therapy in patients with reduced kidney function. Nephrol Dial Transplant. 2024;39:1976–84. 10.1093/ndt/gfae142 38914465

[8] Other’ big complication: how chronic kidney disease impacts on cancer risks and outcomes. Nephrol Dial Transplant. 2023;38:1071–79. https://academic.oup.com/ndt/article/38/5/1071/6516942 10.1093/ndt/gfac011 PMC10157781 35090037

[9] Bonilla MA, Dale D, Zeidler C et al. Long-term safety of treatment with recombinant human granulocyte colony-stimulating factor (r-metHuG-CSF) in patients with severe congenital neutropenias. Br J Haematol. 1994;88:723–30. 10.1111/j.1365-2141.1994.tb05110.x 7529539

[10] Magen D, Mandel H, Berant M et al. MPGN type I induced by granulocyte colony stimulating factor. Pediatr Nephrol. 2002;17:370–2. 10.1007/s00467-002-0847-9 12042897

[11] Nasilowska-Adamska B, Perkowska-Ptasinska A, Tomaszewska A et al. Acute glomerulonephritis in a donor as a side effect of allogeneic peripheral blood stem cell mobilization with granulocyte colony-stimulating factor. Int J Hematol. 2010;92:765–8. 10.1007/s12185-010-0730-6 21120643

[12] Funakoshi Y, Nazneen A, Nakashima Y et al. Possible involvement of G-CSF in IgA nephropathy developing in an allogeneic peripheral blood SCT donor. Bone Marrow Transplant. 2010;45:1477–8. 10.1038/bmt.2010.4 20190844

[13] Arora S, Bhargava A, Jasnosz K et al. Relapsing acute kidney injury associated with pegfilgrastim. Case Rep Nephrol Urol. 2012;2:165–71. 10.1159/000345278 23326257 PMC3542938

[14] Khan TA, Hassan A, Ali ASS et al. IgA. nephropathy presenting as RPGN after G-CSF administration: an interesting case. J Am Soc Nephrol. 2015;26:894. https://www.asn-online.org/education/kidneyweek/archives/KW15Abstracts.pdf (7 February 2026, date last accessed).

[15] Lee JBL, Billen A, Lown RN et al. Exacerbation of IgA nephropathy following G-CSF administration for PBSC collection: suggestions for better donor screening. Bone Marrow Transplant. 2016;51:286–7. 10.1038/bmt.2015.224 26437069

[16] Anupama PH, Abraham G, Koshy P et al. Filgrastim-related acute kidney injury in a male renal transplant recipient. Saudi J Kidney Dis Transplant. 2018;29:739. 10.4103/1319-2442.235196 29970758

[17] Elabd H, Nayak R, Rashid T et al. The Case Acute kidney injury in a 78-year-old man with low-grade B-cell lymphoma. Kidney Int. 2018;93:275–6. 10.1016/j.kint.2017.07.001 29291825

[18] Development of membranoproliferative glomerulonephritis-like glomerulopathy in a patient with neutrophilia resulting from endogenous granulocyte-colony stimulating factor overproduction: a case report. https://link.springer.com/article/10.1186/s12882-018-1049-4 (7 February 2026, date last accessed). 10.1186/s12882-018-1049-4 PMC6172840 30286731

[19] Guffroy B, Ingwiller M, Gavand P-E et al. Flare of IGA glomerulonephritis under G-CSF stimulation regimen for autologous stem cell transplantation in systemic sclerosis. Rheumatology. 2020;59:e33–4. 10.1093/rheumatology/kez662 31953944

[20] Chandra P, Dahiya S, Sanchez-Petitto G et al. Acute glomerulonephritis in a hematopoietic blood stem cell donor. Clin Nephrol—Case Stud. 2021;9:81–86. 10.5414/CNCS110538 34235045 PMC8259466

[21] Angel-Korman A, Leiba A. Filgrastim-induced ANCA-associated glomerulonephritis in the presence of membranous “full house nephropathy”: PO1494. J Am Soc Nephrol. 2021;32:470. 10.1681/ASN.20213210S1470b PMC9415626 36000895

[22] Villegas-Gamas JM, Márquez-Macedo SE, Jiménez-Franco B et al. Proliferative glomerulonephritis with monoclonal IgG deposits triggered by filgrastim in a patient with multiple myeloma. J Nephrol. 2023;36:1209–12. 10.1007/s40620-022-01555-y 36652170

[23] Hattori K, Shimizu R, Tanaka S et al. IgA nephropathy diagnosed as a result of acute exacerbation due to G-CSF administration. CEN Case Rep. 2023;12:270–4. 10.1007/s13730-022-00764-5 36508113 PMC10393928

