# Supplementary material for: Crescentic transformation induced by granulocyte-colony stimulating factor administration—a case report and review of the literature
Source: Clin Kidney J. 2026 Aug 11;19(9):sfag258. doi: 10.1093/ckj/sfag258 (PMC13548867; doi:10.1093/ckj/sfag258)
Supplement: Supplementary file 1 [file sfag258_supplemental_file.docx]

**SUPPLEMENTARY MATERIALS**

**Supplementary Table S1:** Naranjo Adverse Drug Reaction Probability Scale for our patient

| **Parameter** | **Answer** | **Score** | **Comments** |
| --- | --- | --- | --- |
| 1. Are there previous conclusive reports on this reaction? | **Yes** | +1 | Multiple biopsy-proven cases of G-CSF–associated GN, including crescentic GN, are documented in the literature. |
| 2. Did the adverse event appear after the suspected drug was administered? | **Yes** | +2 | AKI and haematuria developed within 24 hours of filgrastim administration. Strong temporal relationship. |
| 3. Did the reaction improve when the drug was discontinued? | **Yes** | +1 | No further filgrastim was given; renal function improved with treatment. |
| 4. Did the reaction reappear upon re-administration? | Not done | 0 | No rechallenge with filgrastim was performed. |
| 5. Are there alternative causes that could have caused the reaction? | **Yes** | −1 | Underlying MGRS with MPGN, possible disease progression, treatment-related immune activation, infection. |
| 6. Did the reaction reappear with placebo? | Not applicable | 0 | No placebo exposure. |
| 7. Was the drug detected in toxic concentrations? | Not applicable | 0 | Not applicable for filgrastim. |
| 8. Was the reaction more severe with increased dose or less severe with decreased dose? | Unknown | 0 | Single-dose exposure only. |
| 9. Did the patient have a similar reaction to the same or similar drugs previously? | No | 0 | No prior exposure documented. |
| 10. Was the adverse event confirmed by objective evidence? | **Yes** | +1 | Biopsy-proven crescentic transformation with clear clinical and laboratory correlation. |

**Total score: 4** (Possible Adverse Drug Reaction)

**Supplementary Table S2:** MEDLINE Search Strategy

Database(s): Ovid MEDLINE(R) ALL 1946 to October 01, 2025
Search Strategy:

| **#** | **Searches** | **Results** |
| --- | --- | --- |
| 1 | exp Granulocyte Colony-Stimulating Factor/ | 17 414 |
| 2 | (granulocyte adj3 colony-stimulating adj3 factor).ti,ab,kf. | 31 458 |
| 3 | (Filgrastim or Neupogen or Pegfilgrastim or Neulasta or Molgramostim or Granix or Eflapegrastim or Argran).mp. | 3753 |
| 4 | G-CSF.ti,ab,kf. | 17 210 |
| 5 | or/1-4 | 43 130 |
| 6 | exp Glomerulonephritis/ | 55 014 |
| 7 | exp Acute Kidney Injury/ | 61 987 |
| 8 | exp *Vasculitis/ | 90 260 |
| 9 | (acute kidney adj5 (injur* or insufficienc* or failure*)).ti,ab,kf. | 51 067 |
| 10 | (Glomerulonephritis or AKI).ti,ab,kf. | 61 469 |
| 11 | or/6-10 | 236 464 |
| 12 | 5 and 11 | 349 |
| 13 | limit 12 to english language | 328 |
| 14 | exp Glomerulonephritis/ and (Glomerulonephritis or AKI).ti,ab,kf. | 22 885 |
| 15 | exp *Glomerulonephritis/ | 43 676 |
| 16 | exp *Acute Kidney Injury/ | 50 210 |
| 17 | (acute kidney adj5 (injur* or insufficienc* or failure*)).ti,kf. | 31 223 |
| 18 | glomerul*.ti. | 44 811 |
| 19 | nephrotoxicit*.ti,kf. | 10 001 |
| 20 | vasculitis*.ti. | 16 943 |
| 21 | or/14-20 | 153 732 |
| 22 | 13 and 21 | 147 |
| 23 | ("36000895" or "23326257" or "27147425" or "34235045" or "35201596" or "31953944" or "36508113" or "30286731" or "12042897" or "10931006" or "36652170").ui. | 11 |
| 24 | 22 and 23 | 11 |
| 25 | 22 not 24 | 136 |
